# GRANDPARENTS' ENTITLEMENTS AND OBLIGATIONS

**DOI:** 10.1111/bioe.12028

**Published:** 2013-05-30

**Authors:** Heather Draper

**Keywords:** grandparents, obligations, entitlements, grandchildren, visitation rights, responsibilities, relationships

## Abstract

In this article, it is argued that grandparents' obligations originate from parental obligations (i.e from the relationship they have with their children, the parents of their grandchildren) and not from the role of grandparent per se, and any entitlements flow from the extent to which these obligations are met. The position defended is, therefore, that grandparents qua grandparents are not entitled to form or continue relationships with their grandchildren. A continuation of grandparent-grandchildren relationships may be in the interests of children, but the grandparental nature of the relationship is not decisive. What counts is the extent to which relationships children have with any adults who are not their parents are is significant to them. Sometimes, however, grandparents become parents or co-parents of their grandchildren. They then gain parental rights, and as such are as entitled, ceteris parius, as any parent to expect their relationship with the child to continue. The issue of grandparents' entitlements can come to the fore when parents separate, and grandparents are unhappy with the access they have to their grandchildren. Grandparents' obligations may become a particular issue when parents die, struggle, or fail to care for their children. This article focuses particularly on these kinds of circumstances.

## Introduction

Grandparents provide a significant amount care for their grandchildren in developed counties. Some of this is care in conjunction with their grandchild's parents: for instance, in Australia they provide 26% of non-parental care.^1^ In the UK grandparents provide the biggest proportion of the informal care used by parents,^2^ consistently around 25%.^3^ Hank et al. report that across Europe the amount of grandparental care varies between 20–40%, with the southern countries tending to provide the higher percentages and the Scandinavian ones the lower, but with significant outliers in both regions.^4^ As well as care in conjunction with parents, some grandparents are also main carers for their grandchildren. In Australia 16,000 grandparents are the main carers of children 0–17.^5^ In the USA figures vary. Livingstone & Parker^6^ suggest that 10% of children live with a grandparent and in 41% of these families the grandparent is the main carer for the child, and in 43% of these cases the grandparent is the sole carer. Timmons and Dye,^7^ reporting on the Census 2000, claim that 3.6% of grandparents live with their grandchildren and 42% of these are ‘providing most of the basic needs for children under 18’.

These figures and the range of involvement grandparents can have with their grandchildren raise at least three ethical questions that will be addressed in this article:

1. To what extent are grandparents obliged to provide care for their grandchildren?^8^ To answer this question, we must also explore what the origins of any proposed obligations might be.
2. Are grandparents who are not willing to be involved in the lives of their grandchildren falling short in their obligations?
3. Are grandparents entitled to be involved in the lives of their grandchildren, and if so how might parental separation affect this entitlement?

Divorce and parental separation are becoming increasingly common in economically developed countries.^9^ This trend has made it important to decide with whom children should live after their parents separate. It is not just parents who may expect access to children post-parental separation; maternal and paternal grandparents (and other relations of the separated parents) may also feel entitled to have regular access to, or time with the children. This can be difficult to achieve if, for instance, parental separation is acrimonious, or the children are living many miles away, or if the grandparents only learn of the existence of the children some years after they are born.

In the USA, where grandparents have the legal right to petition for visitation, courts have ordered visitations with grandparents on a par with those of parents even in circumstances where other court orders are already in place dividing the child's time between parents/other adults.^10^ Policies for access to children post-parental separation in the UK,^11^ Canada,^12^ and Australia^13^ give prominence to the interests of children,^14^ but the positive role that grandparents can play when parents separate is widely recognized across jurisdictions.^15^

## Framing the Questions: Two Ways of Considering the Obligations and Entitlements of Grandparents

There are broadly two ways of considering the obligations and entitlements of grandparents. The first straightforwardly considers the welfare of the child: where involvement with grandparents serves the welfare of the child, then this is a reason to protect and encourage grandparent-grandchild relationships. This formulation is consistent with the paramountcy of the welfare of the child in law and policies in the UK, much of the European Union and Australia, but says more about the rights of children than it does about the entitlements of grandparents. It does not mark out grandparents for special entitlements: the same argument could be used to promote contact between the child and anyone else with whom that child enjoys a beneficial relationship.

The second kind of claim is that grandparents by *virtue of being grandparents* are entitled to form a relationship with their grandchildren. This sort of claim suggests that grandparents can, as it were, reach over the heads of parents to their grandchildren, and may even be able to press a claim for contact when the grandchildren resist (presuming that it is not demonstrably contrary to the child's interests). Such a claim would give weight to the sense that grandparents should be the preferred carers for children if their parents are unable to fulfil their responsibilities, and it may enable grandparents who have no existing relationship with their grandchildren to initiate contact. This is significant since the first kind of argument – the welfare of the child argument – does little to distinguish between grandparents and anyone else. A child may form a significant relationship with any adult – family member or otherwise – the cessation of which might be detrimental; but (parents aside) not just any adult can argue that it may be beneficial to a child to *begin* a relationship with him. If it is thought significant to the welfare of a child to have access to, and a relationship with, grandparents specifically, then this marks out grandparents for special treatment. To this extent, at least some of the sorts of claims made by grandparents appealing to the welfare of the child include an assumption that *as grandparents* they have special entitlements viz their grandchildren. Whilst claims by grandparents will rarely trump the welfare claims of children, they may undermine the authority of parents.^16^^,^^17^

One challenge to a strong presumption of this kind is that not all grandparents want the responsibilities that may accompany it. If the grandparent-grandchild relationship warrants protection (akin to that of the parent-child relationship) then the accompanying responsibilities attach to all grandparents, just as they do to parents. Accordingly, just as parents are open to criticism for failing to step up to the plate of parental responsibility, so would grandparents be criticizable if they failed to be sufficiently ‘grandparent-like’.^18^

This article will attempt to identify the source of grandparents’ obligations, to their grandchildren. It will reject the view that genetic relatedness gives rise to grandparental obligations and suggest instead that the parent-child relationship is the origin of grandparents’ obligations.^19^ Finally, it will consider what entitlements and obligations grandparents might have to continue relationships with their grandchildren.

## Genetic Relatedness

Policy and law in many countries gives special weight to genetic relatedness in assigning parental obligations (and rights). This is so even in countries that protect non-genetic family relationships (those arising, for instance, from adoption, remarriage, or conception using donated gametes). For instance, the European Convention on Human Rights Article 8 protects family life, and although the quality of the relationship with the child is vital to cases brought under the Convention, it seems clear that whilst blood ties (i.e. genetic relatedness) are not necessary to establish family ties, they may be sufficient, especially in regard to wider family.^20^ The conventions tend to use general terms like ‘family relations’, ‘family’ and even ‘parent’ without defining what these mean. In view of this, valuing specifically genetic ties is as much a social norm as it is something required by international agreements, and it is open to question whether genetic relationships ought to be regarded as significant or not.

Among philosophers genetic relatedness has not proved to be a convincing basis for defining parenthood or the source of parental responsibilities. The debate about the origins of parental responsibility extends over a vast literature, of which only a brief outline can be provided here. Daniel Callahan, for example sought to defend ‘the moral seriousness of biological [genetic] fatherhood’ as a ‘natural bond [that] cannot be abrogated or put aside’^21^ so that even post-adoption a biological father retains responsibility for his child should that adoption fail. But his argument rests, in fact, not on biology but the contention that actions voluntarily^22^ undertaken caused the child to exist and create obligations for as long as the child exists. The child, once he exists, is in need of care and nurture, and it is the responsibility of those who voluntarily caused him to exist to supply these things. Callahan's argument does not support obligations flowing from genetic relatedness per se but rather genetic relatedness is evidence of voluntarily causing the needy child to exist. Voluntarily causing is thus the critical notion.

Rivka Weinberg proposes something similar, arguing that the risk that gametes, if combined, will produce a needy child imposes serious responsibilities.^23^ For reasons we will return to, Weinberg also argues that these responsibilities cannot be transferred. Kolers and Bayne^24^ roundly reject the claim that genetic relatedness is necessary and sufficient for parenthood, though they accept – by proposing a pluralistic approach^25^ – that it may be sufficient, and agree that looking for the origin or cause of a child is a relevant factor in determining who bears responsibility for her. Their pluralistic account includes different sorts of cause, including intention. The pertinent question for this article is whether, even if a causal (genetic) chain of some kind is at least sufficient to explain parental responsibility, it can also be used to explain grandparental responsibility.

Parents are not generally regarded as being responsible for the voluntary actions of their (adult) children;^26^ and having a child cannot by itself create a risk that vulnerable children will be brought into being who, *but for* the reproductive choices of their *grand*parents, would not have existed. If this is kind of causal chain were sufficient to generate obligation, why stop at grandparents rather than great grandparents and so forth back through time? Rather than establishing a basis for responsibility, this appeal to genetic relatedness is an invitation to shuffle off responsibility, since everyone can argue that they didn't choose to be born. Moreover, according to the voluntary cause argument, it is the vulnerabilities of children as minors that give rise, to parental obligations. This suggests that the voluntary cause argument only explains parental obligation to their *minor* (vulnerable and needy) children and not their adult ones.^27^ Indeed, the literature on parenthood tends to be confined to the origins of parental responsibilities, and it only addresses the point at which parental responsibilities may end in terms of whether these responsibilities can be meaningfully transferred early in a child's existence (for example by discussing surrogacy, gamete donation or adoption). There is comparative silence on the issue of what responsibilities parents might have towards their adult children, and we will be returning to this issue in the next section.

Callahan considers that the responsibilities of parenthood are such that they can never be transferred: ‘once a father, always a father’.^28^ But not all those who give weight to genetic relatedness when defining parental responsibility agree with him. David Benatar, for instance, argues that ‘people have a presumptive responsibility for rearing children who result from their gametes’ because they ‘bear responsibility for their reproductive conduct’ insofar as this is an expression of their reproductive autonomy.^29^ Nonetheless, he accepts that parental responsibilities can be transferred, but they must be transferred responsibly not lightly. For the purposes of defining grandparenthood, it may be sufficient to accept that parental responsibilities clearly *can* be transferred (after all, adoptive parents without doubt acquire parental responsibility) even if we leave aside the question of whether they *ought* ever to be. Granted it is possible to transfer parental responsibility i.e. for a parent to cease to be a parent, it is not clear how that person's parents can, *ceteris paribus*,^30^ claim to be grandparents with grandparental rights or obligations.

On the contrary, given that parental responsibility has been transferred, the proper conclusion is that the parents of the parents by adoption become the grandparents of an adopted child. Assuming that gamete donation represents a similar transfer of responsibility, and given that no relationship is likely to have formed between the gamete donor's parents and the child, the grandparents of the resulting children will once again be the parents of the gamete recipients, not the parents of the gamete donor. Indeed, to argue otherwise in either the case of adoption or gamete donation undermines the legitimacy of the parents in both cases.

Now, it might be argued that when a parent transfers parental responsibility they do not cease to be a parent; they just cease to have parental responsibility. Accordingly, the genetic grandparents remain grandparents in some sense. In what sense such a label is morally significant or gives rise to responsibilities is not clear, however. If terms like ‘parent’ or ‘grandparent’ merely describe genetic connections rather than ascribing a role, they are devoid of normative content. A genetic family tree does nothing to explain why those on it might have special obligations or entitlements in relation to the children at the tip of its branches. Further argument is needed to explain why being genetically related to a greater rather than lesser degree generates obligations, especially where the elements of voluntary cause are clearly not applicable (which applies as much to aunts, cousins or other members of the wider family as it does to grandparents).

In conclusion, genetic relatedness as the source of parental responsibilities has intuitive appeal, but scratch the surface and factors such as voluntarily causing or risk-taking are required to explain parental responsibilities and obligations. These factors are not operative in the case of grandparents, and so do not explain why grandparents might have obligations to grandchildren. If grandparents do have obligations, some alternative explanation for them needs to be found.

## Grandparenting as an Extension of Parenting

Adoption, using donated gametes to create children, step-parenting and blended families all challenge the significance of genetic ties and voluntary cause as the explanation for familial obligation. Instead of genes, actions – commitment to and performance of parental responsibilities generated by a child's needs – may confer parental status, and in turn parental rights.^31^ Parental love is also a significant element of parenting. Weinberg considers that loving a child is a parental obligation and it is for this reason that parental responsibilities cannot be transferred: someone cannot be asked to love another in one's place.^32^ Whilst I agree with her that love is a vital component of parenting, I struggle with the notion that one can be obligated to love another, since it is far from obvious that love is a product of the will. Again, it is clear that parents who adopt children or gain them by means of gamete donation can in fact love them as deeply as any other parent. So, perhaps what we should conclude is not that parental responsibility cannot be transferred, but rather that it ought not to be; that with rare exceptions,^33^ the willing transfer of parental responsibility is a failure of parenting. We may go further and say that for parents the willing transfer of parental responsibility is normally unconscionable *because* they love their children. And we might also say that parental love and the fulfilment of parental responsibilities for a needy child defines one as a parent.

Parental responsibility is not necessarily limited to the period before legal majority. In short, parenting lasts as long as one is able to continue. One reason that parenting does not end at legal majority is that parents do not stop loving their children just because they have reached adulthood. Parental love and responsibility take different forms over the child's life-time according to the child's changing needs. This means that although parents are not *responsible* for the consequences of their children's achievements and failures, they may have continuing obligations to help when the consequences are adverse or burdensome.

We can now begin to see the source of grandparental obligations in their own parental obligations. Parents love their children, even when those children are adults and have children themselves. Indeed, it is when their children have children that they may need assistance. Childrearing is hard work, sometimes relentlessly hard work, which often has to be performed alongside paid work. Here, then, are two reasons why grandparents might be obliged to become involved in the lives of their grandchildren; the burdensome nature of childcare for the grandchildren's parents, and the significance of the grandchildren to their parents. Let us take the latter first.

The nature of parental love is such that the interests of the parents and child become intertwined and ‘selflessness and self-interest coincide’.^34^ That which is significant to children, matters to their parents.^35^ In the case of grandparents their children's children ought to be important to them just because they are important to their children. Simply put, the person who unreasonably refuses to show any interest in her grandchildren – even though they are clearly important to her child – is failing to be a good parent rather than failing to be a good grandparent. Equally, the person who unreasonably interferes in the relationship between her daughter and her daughter's child is failing to be a good parent because she is meddling in her daughter's affairs rather than supporting her. The extent to which grandparents are involved in the lives of their grandchildren will depend on the extent to which they are involved in the lives of their children. This may in part be reflected in how they help to shoulder the burden of childrearing.

The obligation to help with the burdens of childrearing will depend on a variety of factors ranging from the extent to which it is a burden to the parents (which may depend on their own means and dispositions) to the extent to which the grandparents are themselves able to help. It will also include the costs to the grandparent of helping: some grandparents want nothing more than to be involved in the lives of their grandchildren; for others, being released from their own childcare responsibilities is an opportunity to achieve the ambitions that were incompatible with those responsibilities. The costs for the latter grandparents may be sufficiently weighty to outweigh the obligation to help as the obligation to meet one's children's needs lessens as one's children mature and are able to meet their own needs. As a child becomes less vulnerable and in need of nurture, the obligation to prioritize her needs lessens in the day-to-day life of the parent, even though it remains significant. It is not unreasonable, therefore, for parents to prioritize their own life plans ahead of the day-to-day plans of their adult children, and this may include, for example, not taking on full-time day-care responsibilities for grandchildren, even though this would help their parents. On the other hand, in an emergency or in the case of other forms of extreme need, the needs of adult children sometimes *have* to take priority. It may be unreasonable to demand that a grandparent relocates away from her own job and social network to care for grandchildren, and equally unreasonable to refuse an extended visit periodically, or in an emergency, or to communicate with grandchildren at a distance if this matters to the adult child. On the other hand, constant unwelcome interference may be a continuation of a pattern of poor parenting, whereas unsolicited advice to prevent a bad outcome might be an unpopular but necessary part of parenting an older child, even an adult one. But these judgements are similar to those that are routinely left to parents and will be handled differently by different parents with different degrees of generosity and success.

We are now in a position to answer two of the three questions posed at the start of this article, namely whether grandparents have an obligation to provide care for their grandchildren and whether those who do not are failing in this obligation. The case for a *prima facie* obligation has been made. The extent to which the failure to meet this obligation is a failure depends largely on the costs to the grandparent of meeting the obligation relative to the circumstances of the parent. This prima facie obligation is, however, a *parental* obligation not a *grand*parental obligation. It offers an explanation for the origins of grandparents' obligations but may not give full account of the obligations in play.

Relationships formed with grandchildren can generate direct obligations to the grandchildren themselves rather than their parents. But it is not clear that what provides the normative element here is a distinctively grandparent-grandchild relationship. A general argument can be made for the wrongness of giving children false expectations, or breaking promises, or being unreliable and so forth. Children are especially vulnerable as they are likely to be more trusting and less canny than adults, and this heightens the general obligation to be truthful, keep promises and so forth. Any adult in an especially close relationship with a child will have similar obligations of this kind. These are the obligations of adults to children they interact with and care about. They are not distinctively the obligations of family members, and this being so, there may be no *distinctively* grandparental obligations at all, but only caring adult obligations discharged by grandparents.

## Grandparents Who are Also Parents of Grandchildren

Grandparents may be thought to have increased obligations to care for their grandchildren if their parents are unable (perhaps due to death or imprisonment) or unwilling to discharge their parental obligations to their children.

When a parent of a minor dies, the surviving grandparent may want to ensure that their own child's hopes for and obligations to the grandchild are fulfilled. In this respect, becoming a parent is unlike other life-interests such as a career or sporting ambition that dies with the person. The product of this life-interest is a needy child who is, if anything, needier as a result of his parent's death. The strength of the obligations here may depend on the circumstances, for example, on whether the child has another living and engaged parent. If not, although the grandparent's sense of obligation may be acute, what happens next must also be decided by the welfare of the child and what best meets his needs. Depending on the grandparents' own circumstances these may or may not be met by taking the child into their home and rearing him. If the grandchild has another competent parent, the grandparents may still be obliged to shoulder some of the burden and all parties may benefit from the continuation good grandparent/grandchild relationships and solicited help with the burdens of child-rearing. There may also be parents of the remaining parent who should share this burden. Arguably, the more people there are who ought to share the burden, the less the burden is and therefore the greater the obligation to do one's share, for as the weight of the burden decreases the more difficult it is to justify opting out of shouldering it.

In the case where parents are struggling or unwilling (as opposed to unable) to fulfil their parental responsibilities, the grandparents may feel that they ought to fulfil them in their place. Leaving to one side issues of reputation or family name and honour, this might be akin to parents wanting to clear an adult child's debts or offer compensation for some harm their adult child has caused. However, as parents have no *obligation* to make good their adult child's wrongdoing, contribution beyond that owed by their parental obligations would be supererogatory. Again, the interests of the child should be decisive in determining the extent to which the grandparent should take over a childrearing role. In the USA, where the most data seems to be have been collected, the record of grandparents as sole carers is not one of overwhelming success. The grandchildren are disproportionately likely to live in poverty, suffer mental illness, experience behavioural problems and contract sexually transmitted disease, for instance.^36^ This may, however, be yet more evidence of the effect of the poverty of older people being combined with child poverty,^37^ and other general disadvantages felt by children born into chaotic families, rather than evidence that grandparents do a disproportionately poor job of bringing up their grandchildren. Moreover, grandparents may do a better job of childrearing even in these circumstances than any of the available alternatives.

As these examples illustrate, grandparents' contribution to childcare may assume the proportions of a full-scale parental contribution. In these cases, grandparents might justifiably regard themselves as sole parents or co-parents, with all the accompanying rights of parents. This is especially pertinent to the third question raised in the introduction, whether grandparents can be entitled to continued relationships with their grandchildren.

When considering what entitlements grandparents might have in relation to their grandchildren, it is necessary to distinguish between substantial care given to help parents of the kind also given by people who are not relations of the children, and care that replaces or substitutes for parental care. Many parents are unable to provide full time care for their young children (especially parents who need to take paid employment) and the majority of children spend substantial amounts of their day during term time in school, where besides being obliged to provide education, the school is obliged to provide personal care in proportion to the age and circumstances of the child. This care may be considerable in boarding schools and summer camps, for example. Significant relationships may develop between children and their childminders, nannies, au pairs, teachers, school staff (teachers, counsellors and matrons) but they are not regarded as so significant that any of the above are *entitled* (or obliged) to continue with such relationships when they are brought to an end either through circumstance or parental will. Sometimes the grandparental contribution is no greater than, and deserves no more recognition than, that of the responsible but unrelated adult.

On the other hand, where grandparents provide sole care over an extended period and in the absence of parental input, fulfilling the responsibilities of parenthood, then grandparents should be afforded the associated rights of parenthood, including *ceteris parius* the right to continue to rear their child. But this is a *parental* right, not a *grand*parental right. In these circumstances, grandparents have a special claim because they have become parents and their claims should be considered on a par with anyone else who also claims to be the child's parent, with the interests of the child being the decisive factor in both formal and informal dispute resolution.

Grandparent-parents face a particular personal dilemma if they come into conflict with co-parents who are their children. Parenting, even in the case of adult children, remains associated with protecting and promoting the interests of the child. It might therefore be assumed that grandparents are generally obliged not to contest custody of grandchildren where the other party to the conflict is their own child. The potential dilemma for the grandparent-parent is not, however, between their obligations to their child and their grandchild, but their competing obligation to their two children, the latter of whom is also a grandchild. This kind of conflict is familiar to all those who are the parent of more than one child, and it is not obvious that such tensions must always be resolved in favour of the oldest, most vocal and least vulnerable child. Shared-care and co-operative co-parenting may be the most appropriate outcome.

## Concluding Remarks

Grandparental obligations arise from parental obligations. Some grandparents in fulfilling their obligations to the parents of grandchildren develop strong, independent relationships with their grandchildren. But it is not obvious that these relationships are significant because they are made with grandparents. Children can and do develop significant relationships with adults who are not related to them. The significance of the relationship to the child is more pertinent to determining whether or not the relationship warrants protection. In this respect, that a child has *a* relationship with a *grandparent* is not sufficient to regard that relationship as a special one.

There is evidence that grandparents can enhance the welfare of their grandchildren.^38^ In order for grandchildren to benefit from such relationships, they have to be built up over time. Such relationships can be disrupted when parents separate. Willing grandparents may find it difficult to establish and maintain beneficial relationships with their grandchildren, and this may be to the detriment of all concerned. Equally, children with separated parents may already have their time and energies divided between two different households and families, and, as they grow older, also have additional competing demands on their times from friends and other social networks. This may make it difficult for them to find time to attend to the multitude of relationships in their different wider families, including those with grandparents.^39^ These competing factors ought to be given due consideration by those seeking greater access to these children, especially those purporting to be representing a child's interests.

Visitation rights in the USA resulted from petitioning by grandparent lobby groups. The success of the petitions no doubt had something to do with the power of the ‘grey’ vote, but it is also connected to some intuitive sense that grandparents have a special status in the lives of their grandchildren that has gone unchallenged. This intuition is not confined to the USA. In the UK, for example, the Centre for Social Justice concluded that:

… grandparents seeking contact [with their grandchildren] should not be placed in the same legal position as other extended family members or stepparents to the family who need leave to apply to the court.^40^

But our intuitions can be mistaken. Following the Family Justice Review,^41^ the UK government decided that grandparents should continue to apply for leave of court to petition for access.^42^ In Australia, the Family Law Amendment (Share Parental Responsibility) Act 2006 (Cth) states that ‘children have the right to spend time on a regular basis with, and communicate with, both parents and other people significant to their care, welfare and development (such as grandparents and other relatives). [s60B(2)(b)]. In both Australia and the UK, then, legislators have resolved the question of grandparental access by acknowledging that grandparents may form special relationships without assuming that they are special *because* they are made with grandparents.

## Footnotes

1 Australian Bureau of Statistics. *Childhood Education and Care Australia June 2011* cat. No. 4402.0. Available at: http://www.abs.gov.au/ausstats/abs@.nsf/Products/4402.0∼June+2011∼Main+Features∼Main+features?OpenDocument [accessed 13 Aug 2012].

2 P. Smith et al. 2010. Childcare and Early Years Education Survey of Parents. Official Statistics Release OSR 12/2012 Research Report DFE-RR221 Available at: http://www.education.gov.uk/rsgateway/DB/STR/d001073/osr12-2012main.pdf [accessed 13 Aug 2012].

3 J. Statham. *Grandparents Providing Care; a Briefing Paper*. Department for Education November 2011 Available at: http://www.education.gov.uk/publications/RSG/EarlyYearseducationandchildcare/Page1/CWRC-00083-2011 [accessed 13 Aug 2012].

4 I. Buber & K. Hank. Grandparents Caring for their Grandchildren: Findings from the 2004 Survey of Health, Ageing, and Retirement in Europe. J Fam Issues 2009; 30(10): 53–73.

5 Australian Bureau of Statistics *Family Characteristics Australia 2009-10* cat. No. 4442.0 Available: http://www.abs.gov.au/AUSSTATS/abs@.nsf/Latestproducts/4442.0Main%20Features22009-10?opendocument&tabname=Summary&prodno=4442.0&issue=2009-10&num=&view [Accessed 13 Aug 2012].

6 G. Livingstone & K. Parker. Since the Start of the Great Recession, More Children Raised by Grandparents. Pew Social and Demographic Trends 9^th^ September 2012. Available at: http://www.pewsocialtrends.org/2010/09/09/since-the-start-of-the-great-recession-more-children-raised-by-grandparents/ [accessed 13 Aug 2012].

7 T. Timmons & J.L. Dye. Grandparents Living with Grandchildren: 2000 October 2003 United State Census Bureau. Available at: http://www.census.gov/prod/2003pubs/c2kbr-31.pdf [accessed 13 Aug 2012].

8 The European survey reported by Hank & Buber, *op. cit.* note 4, also asked respondents whether they thought grandparents had a duty to help their grandchildren's parents with child care: the majority agreed (regardless of whether they were providing any care) but the response varied according to country. In Netherlands and Denmark only around half of even those caring for grandchildren thought they had a duty to do so.

9 For instance in the UK, 26% of children living in families live with only one parent and 92% of lone parents are women. (Office for National Statistics Families and Households 2001–2011 published in January 2012. Available at: http://www.ons.gov.uk/ons/dcp171778_251357.pdf [accessed 13 Aug 2012]. In Australia 22% of children (under the age of 15 years) were living with one parent in 2004–2006 (Australian Bureau of Statistics Australian Social Trends, 2007 4102.0 One Parent Families. Available at: http://www.abs.gov.au/ausstats/abs@.nsf/0/F4B15709EC89CB1ECA25732C002079B2?opendocument#HOW%20MANY%20ONE-PARENT%20FAMILIES%3F [accessed 24 Aug 2012]. In the USA, 32% of families with children were single-parent families (26% headed by women and 6% headed by men) (J. Fields. 2003. *America's Families and Living Arrangements 2003*. Current Population Reports: 20–553. US Census Bureau, Washington, DC). In Canada in 2006, 16% of children lived in single parent families (80% of which were headed by women) Statistics Canada Census Snapshot of Canada – families. 2006 Available at: http://www.statcan.gc.ca/pub/11-008-x/2007006/article/10380-eng.htm [accessed 2 Aug 2012].

10 L.C. Nolan. Beyond Troxel: the Pragmatic Challenges of Grandparent Visitation Continue. Drake Law Review 2001–2002; 50: 267.

11 Children Act 1989. Available at: http://www.legislation.gov.uk/ukpga/1989/41/contents [accessed 22 Aug 2012].

12 Divorce Act 1985 Sec 16(8) Available at: http://laws-lois.justice.gc.ca/PDF/D-3.4.pdf [accessed 24 Aug 2012].

13 Family Law Amendment (Share Parental Responsibility) Act 2006. Available at: http://www.comlaw.gov.au/Details/C2006A00046 [accessed 4 Sept 2012].

14 Although, as James Dwyer notes, giving the interests of the child paramountcy does not ensure that other interests will not be considered in situations where there is little to choose between two solutions in terms of the child's interests. He suggests that few jurisdictions – Canada being one – focus *solely* on the interests of the child in parental disputes, for instance. J.G. Dwyer. 2006. The Relationship Rights of Children. New York: Cambridge University Press.

15 See for instance L. Qu *et al*. Grandparenting and the 2006 Family Law Reforms. Fam Matters 2011; 88: 42–50.

16 Much of the debate in the USA, especially post-Troxel is over whether grandparent visitation undermines parental authority / parental or family autonomy: ‘the Troxel Court recognized and affirmed a parent's right to the care, custody, and control of a child, absent a showing of unfitness or failure to protect the child's welfare.’ D.R. Victor & K.L. Middleditch. Grandparents Visitation: A Survey of History, Jurisprudence, and Legislative Trends across the United States in the Past Decade. J Amer Acad Mat Law 2009; 22: 391. See also, for example, L.C. Nolan. Honor thy Father and thy Mother: But Court-ordered Visitation in the Intact Family? BYU J Pub L 1993–1994; 8: 51; T. Neilson & R. Bucaria. Grandparent Custody Disputes and Visitation Rights: Balancing the Interests of the Child, Parents, and Grandparents Utah L Rev 2009: 569.

17 Note that this is not a claim about some right to *become* a grandparent: clearly parents cannot insist that their children become parents in order that they can have a relationship with the resulting grandchildren.

18 No jurisdictions force parental responsibilities (beyond the financial) on unwilling parents, even if meaningful contact is in the interest of the child, (See Dwyer, *op. cit.* note 14) so it seems unlikely that even those jurisdictions where grandparents have, for example visitation rights, would require unwilling grandparents to be regularly visited by a grandchild even if this was in the child's interests. Nonetheless, the legal position here is different from the moral one: it is possible to call attention to moral failure without necessarily implying that good behaviour should be compelled.

19 I am not be suggesting that this is the only origin of obligations that people who are grandparents may have. For example, if I undertake to care for my daughter's children whilst she is working away from home I am obliged to do so. But the origin of this obligation is not that she is my daughter, but rather that I voluntarily undertook to do so. I am also obliged to care for her children if, on hearing their cries of distress, I discover that she has left them at home alone, but again, the origin for this obligation is that they are vulnerable and in need of care. The focus of this article is the origins of obligations that are thought to arise from being a grandparent, not those that arise in other ways.

20 See A. Bainham. 2003. Contact as a Right and Obligation. In A. Bainham, B. Lindley, M. Richards & L. Trinder, eds. Children and their Families: Contact, Rights and Welfare. Oxford: Hart Publishing, ch.5.

21 D. Callahan. 1996. Bioethics and Fatherhood. In L. May, R. Strikwerda & P.D. Hopkins, eds. Rethinking Masculinity: Philosophical Explorations in the Light of Feminism. 2nd Edn. Maryland: Rowman and Littlefield Publishers, Inc: 163.

22 The is also debate over what counts as voluntary and the impact of deception (e.g. sperm banditry in relation to sperm) on voluntary action.

23 R. Weinberg. The Moral Complexity of Sperm Donation. Bioethics 2008; 22(3): 166–178.

24 A. Kolers & T. Bayne. ‘Are You My Mummy?’ On the Genetic Basis of Parenthood. J Appl Philos 2001; 18(3): 273–285.

25 T. Bayne & A. Kolers. Towards a pluralistic account of parenthood. Bioethics 2003; 17(3): 221–242.

26 For the purposes of this article I will leave aside the question of whether parents are responsible for children that result from pregnancies of minor children. Even if such parents are then responsible for their grandchildren, this is affects only a subset of grandparents and not is an argument that is applicable to grandparents generally.

27 Save *perhaps* those cases where the adults have been vulnerable from birth or since they were minors. Here the question might be whether this burden is solely laid on the shoulders of parents but should be shared by the state.

28 Callahan, *op. cit.* note 21, p. 163 – but this could be generalized to once a parent (as evidenced by genetic related through the VC argument) always a parent

29 D. Benatar. The Unbearable Lightness of Bringing into Being. J Appl Philos 1999; 16(2): 174.

30 Things might not be equal if they already have a substantial relationship with child who is capable of understanding meaningful relationships.

31 See, e.g. Chapter 3 of Adam Swift and Harry Brighouse's forthcoming book on family values – earlier ideas for which can be found in H. Brighouse & A. Swift. Parents' Rights and the Value of the Family. Ethics 2006; 117(1): 80–108.

32 Weinberg, *op. cit.* note 23, p. 174.

33 I agree with Weinberg that one good example of this might be ‘I loved you so much that I released you to a family more capable of caring for you’ motivation for adoption. Ibid:175.

34 H. Frankfurt. 2004. *The Reasons of Love*. Princeton: Princeton University Press: 62.

35 Of course, what matters to one's children might be repugnant to their parents. The view that parents should engage with that which matters deeply to their children does not necessary require parents to embrace views and practices that their children hold dear: one might take them seriously by engaging in serious and respectful disagreement.

36 O.W. Edwards & A.P. Daire. School Age Children Raised by their Grandparents: Problems and Solutions. J Instr Psych 2006; 33(2). Available at: http://www.freepatentsonline.com/article/Journal-Instructional-Psychology/148367616.html [accessed 4 Sept 2012]; G.C. Smith & P.A. Palmieri. Risk of Psychological Difficulties of Children Raised by Custodial Grandparents. Psychiat Serv 2007; 58(10): 1303–1310.

37 J. Grigg. 2009. For Grandparents Plus and EHFC *The Poor Relation: Grandparental Care where Older People's Poverty and Child Poverty Meet*. Grandparents Plus: London. Available at: http://www.grandparentsplus.org.uk/wp-content/uploads/2011/03/The-Poor-Relation-interim-report.pdf [accessed 4 Sept 2012]; L.A Baker, M. Silverstein & N.M. Putney. Grandparents Raising Grandchildren in the United States: Changing Family Forms, Stagnant Social Policies. J Soc Soc Pol 2008; 7: 53–69. Available at: http://www.ncbi.nlm.nih.gov/pmc/articles/PMC2888319/ [accessed 4 Sept 2012 ].

38 S. Attar-Schwartz et al. Grandparenting and adolescent adjustment in two-parent biological, lone-parent, and step-families. J Fam Psychol 2009; 23(1): 67–75.

39 And it is worth noting that if the number of parents a child has is extended (e.g. to include step-parents) the number of potential grandparents may also increase.

40 Centre for Social Justice. 2009. Every Family Matters: an in-depth review of family law in Britain. London: Centre for Social Justice, p. 30. Available at: http://www.centreforsocialjustice.org.uk/client/downloads/WEB%20CSJ%20Every%20Family%20Matters_smallres.pdf [accessed 22 Aug 2012].

41 Family Justice Review Panel. 2011. *The Family Justice Review Final Report* Crown Copyright: London. Available at: http://www.justice.gov.uk/downloads/publications/moj/2011/family-justice-review-final-report.pdf [accessed 22 Aug 2012].

42 Ministry of Justice and Department for Education. 2012. *Government Response to the Family Justice Review: a system with children and families at its heart*. Crown Copyright: London. Available at: https://www.education.gov.uk/publications/eOrderingDownload/CM-8273.pdf. [accessed 22 Aug 2012].

